# Four MicroRNAs, miR-13b-3p, miR-278-5p, miR-10483-5p, and miR-10485-5p, Mediate Insecticide Tolerance in *Spodoptera frugiperda*

**DOI:** 10.3389/fgene.2021.820778

**Published:** 2022-01-21

**Authors:** Yuanxue Yang, Yun Zhang, Aiyu Wang, Ailing Duan, Chao Xue, Kaiyun Wang, Ming Zhao, Jianhua Zhang

**Affiliations:** ^1^ Institute of Industrial Crops, Shandong Academy of Agricultural Sciences, Jinan, China; ^2^ Department of Plant Protection, Shandong Agricultural University, Taian, China

**Keywords:** *Spodoptera frugiperda*, insecticides, miRNA-13b-3p, miRNA-278-5p, miRNA-10483-5p, miRNA-10485-5p

## Abstract

*Spodoptera frugiperda* is the world’s major agricultural pest and has the distinctive features of high fecundity, strong migratory capacity, and high resistance to most insecticides. At present, the control of *S. frugiperda* in China relies mainly on the spraying of chemical insecticides. MicroRNAs (miRNAs) are a class of small, single-stranded, non-coding RNAs and play crucial regulatory roles in various physiological processes, including the insecticide resistance in insects. However, little is known about the regulatory roles of miRNAs on the resistance of *S. frugiperda* to insecticides. In the present research, the miRNAs that were differentially expressed after cyantraniliprole, spinetoram, and emamectin benzoate treatment were analyzed by RNA-Seq. A total of 504 miRNAs were systematically identified from *S. frugiperda*, and 24, 22, and 31 miRNAs were differentially expressed after treatments of cyantraniliprole, spinetoram, and emamectin benzoate. GO and KEGG enrichment analyses were used to predict the function of differentially expressed target genes of miRNAs. Importantly, ten miRNAs were significantly differentially expressed among the treatments of three insecticides. miR-278-5p, miR-13b-3p, miR-10485-5p, and miR-10483-5p were significantly downregulated among the treatments of three insecticides by RT-qPCR. Furthermore, the overexpression of miR-278-5p, miR-13b-3p, miR-10485-5p, and miR-10483-5p significantly increased the mortality of *S. frugiperda* to cyantraniliprole and emamectin benzoate. The mortality was significantly increased with spinetoram treatment after the overexpression of miR-13b-3p, miR-10485-5p, and miR-10483-5p. These results suggest that miRNAs, which are differentially expressed in response to insecticides, may play a key regulatory role in the insecticide tolerance in *S. frugiperda*.

## Introduction

MicroRNAs (miRNAs) are a class of small, single-stranded, non-coding RNAs of approximately 18–25 nucleotides length (Ambros, 2004; Bartel, 2004). miRNAs are derived from the stem-loop RNA precursors (pre-miRNAs) and are widely found in animals, plants, bacteria, and viruses (Lee et al., 2004). miRNAs have been shown to be involved in posttranscriptional regulation of mRNAs by forming silencing complexes through complementary forms of exact or incomplete nucleotide matches with target messenger RNAs (mRNAs) (Lee et al., 2004). Previous studies have shown that miRNAs account for 1–5% of all genes in animals and that 20–30% of genes are regulated by miRNAs (Stark et al., 2005). A single miRNA can regulate multiple target genes and is involved in a number of complex regulatory pathways (Tsai et al., 2009; Salvi et al., 2019).

In arthropods, a growing number of research studies have indicated that miRNAs play crucial roles in physiological and developmental pathways, such as metamorphosis, embryogenesis, molting, reproduction, immunity, and wing development (Etebari and Asgari, 2013; Lucas et al., 2015a; Lozano et al., 2015; Zhang et al., 2016; Ling et al., 2017; Song et al., 2018; Li et al., 2021a; Shang et al., 2020). Recently, miRNAs have been shown to be involved in the metabolism of plant toxin and the insecticide resistance in different insects. For example, miR-4133-3p regulates gossypol and tannic acid detoxification *via* targeting *cytochrome P450 4CJ1* (*CYP4CJ1*) in *Aphis gossypii* (Ma et al., 2019). In *Nilaparvata lugens*, modulating the expressions of miRNAs novel-85 and novel-191 significantly altered the susceptibility of *N. lugens* to nitenpyram by targeting *CYP6ER1* and *carboxylesterase 1* (*CarE1*) (Mao et al., 2021). In addition, the microinjection of miR-7a or miR-8519 mimics in *Plutella xylostella* decreases the expression of the *ryanodine receptor* and significantly increases the susceptibility of *P. xylostella* to chlorantraniliprole (Li et al., 2015a).

*Spodoptera frugiperda* is native to North America, South America, and subtropical regions and feeds on a variety of crops including corn, soybeans, cotton, and rice (Gouin et al., 2017). Two biotypes, the “corn-strain” and “rice-strain”, have been identified for *S. frugiperda* (Hafeez et al., 2021). *S. frugiperda* has strong migratory capacity, first found in Central and Western Africa in 2016, moving into India in 2018, and invading China in January 2019 (Junior et al., 2012; Goergen et al., 2016; Early et al., 2018; Li et al., 2020). *S. frugiperda* migrates to a new location, and resistant alleles persist in resistant populations (Arias et al., 2019). *S. frugiperda* is now resistant to a variety of chemical insecticides, mainly including organophosphates, carbamates, pyrethroids, and other traditional insecticides (Yu and Jr, 2007; Carvalho et al., 2013). Recent studies have shown that *S. frugiperda* has also developed varying degrees of resistance to new chemical insecticides such as diamides, spinosyns, and benzoylureas (Nascimento et al., 2016; Bolzan et al., 2019; Lira et al., 2020).

A part of miRNAs has been identified in *S. frugiperda*, and their functional study mainly focuses on antiviral immune defense and adaptive evolution (Moné et al., 2018; Karamipour et al., 2019), but the systemic identification and functional analysis of miRNAs involved in insecticide resistance are still unknown. In this research, the expression profiles of miRNAs in *S. frugiperda* have been investigated based on high-throughput sequencing of the small RNA library. The differentially expressed miRNAs related to the tolerance of cyantraniliprole, spinetoram, and emamectin benzoate were systematically detected. More importantly, the effects of miR-278-5p, miR-13b-3p, miR-10485-5p, and miR-10483-5p on the susceptibility of *S. frugiperda* to insecticides were verified.

## Materials and Methods

### Insects

The *S. frugiperda* population was collected from Tancheng (Shandong, China) in 2019 and maintained in the laboratory. *S. frugiperda* was provided by Prof. Xingyuan Men (Shandong Academy of Agricultural Sciences, Jinan, China) and reared in an illumination incubator at 26 ± 1°C with 60–70% humidity and 16 h light:8 h dark photoperiod. *S. frugiperda* was fed on artificial diet which was reported in the previous research (He et al., 2021).

### Insecticides and Chemicals

Cyantraniliprole technical (98.00%) and spinetoram technical (85.80%) were purchased from Sigma-Aldrich (St. Louis, MO, United States). Emamectin benzoate technical (95.00%) was provided by Shandong Lukang Biopesticides Co. Ltd (Dezhou, China). Acetone (reagent grade) and Triton X-100 were purchased from Sigma-Aldrich (St. Louis, MO, United States).

### Toxicity Bioassay

Cyantraniliprole, spinetoram, and emamectin benzoate were dissolved in acetone and diluted to five concentrations of 0.04, 0.2, 1, 5, and 25 mg L^−1^ in the distilled water containing 0.05% Triton X-100. The distilled water, containing 0.05% Triton X-100 and 1% acetone, was used as the control. The toxicity bioassay was performed by the method of leaf-dipping (Fouad et al., 2016). Maize leaves were cut into small pieces of 20 mm long and immersed in each concentration of insecticides for 15s. Third-instar *S. frugiperda* was placed in the air-drying maize leaves. A total of twenty insects were treated in each concentration, and each concentration was repeated three times. All insects were reared at 25 ± 1°C with 50–70% humidity and 16 h light:8 h dark photoperiod. The mortality was checked after treatment in 24 h.

### Sample Collection

Third-instar *S. frugiperda* was treated with cyantraniliprole, spinetoram, and emamectin benzoate at doses of LC_50_ by the leaf-dipping method. After 24 h, the survival insects were collected, and the distilled water, containing 0.05% Triton X-100 and 1% acetone, was used as the control. Each treatment contained 20 insects and was repeated three times. *S. frugiperda* was frozen in liquid nitrogen for RNA extraction.

### RNA Extraction, Illumina Sequencing, and Bioinformatics Analysis

The total RNA was extracted by TRIzol reagent (Invitrogen, United States) following the manufacturer’s procedure. The total RNA quantity and purity were analyzed using a Bioanalyzer 2,100 (Agilent, United States) with RIN number >7.0. Approximately, 3 μg of total RNAs were used to prepare a small RNA library according to the protocol of TruSeq Small RNA Sample Prep Kits (Illumina, United States). The single-end sequencing (50 bp) was performed by an Illumina HiSeq 2,500 at the LC-BIO (Hangzhou, China) following the vendor’s recommended protocol. The adapter dimers, junk, low complexity, common RNA families (rRNA, tRNA, snRNA, and snoRNA), and repeats were removed by ACGT101-miR (LC Sciences, Houston, Texas, United States). In order to identify known miRNAs and novel 3p- and 5p-derived miRNAs, the unique sequences with a length of 18–26 nucleotides were mapped to specific species precursors in miRBase 22.0 by BLAST. The unmapped sequences were BLASTed the specific genomes, and the hairpin RNA structures containing sequences were predicated from the flank 80 nt sequences using RNAfold software (http://rna.tbi.univie.ac. at/cgi-bin/RNAfold.cgi). The differential expression of miRNAs was analyzed by ANOVA based on normalized deep-sequencing counts. The different expression analysis of miRNA was selected with log2 ^(fold change)^ > 1 or log2 ^(fold change)^ < −1 and with statistical significance (*p* < 0.05 or *p* < 0.01). The target genes of differentially expressed miRNAs were predicted by two computational target prediction algorithms (TargetScan 50 and miRanda 3.3a), and the 3-untranslated region (UTR) of the candidate target sequences was used to miRNA target prediction. The data predicted by both algorithms were combined, and the overlaps were calculated. The Gene Ontology (GO) terms and Kyoto Encyclopedia of Genes and Genomes database (KEGG) pathway of these miRNA targets were also annotated.

### Quantitative Real-Time PCR

The relative miRNA expression was analyzed by the quantitative real-time PCR (RT-qPCR). PrimeScript™ RT reagent kit with gDNA Eraser (Takara, Japan). It was used to synthesize the cDNA from 1 µg of total RNA. The quantitative PCR reaction was completed using an iQ™5 Multicolor Real-Time PCR detection system (Bio-Rad, United States) with the SYBR PrimeScript™ RT-PCR Kit (Takara, Japan). The U6 snRNA was used as an endogenous control to normalize the expression levels of miRNAs (Zhang et al., 2021a). The 2^−∆∆Ct^ method was used to calculate the relative expression levels of miRNAs (Livak and Schmittegen, 2001). The RT-qPCR reaction included three independent technical and biological replications. The RT-qPCR primers are listed in Supplementary Table S1.

### Tissue Expression Profile of miRNAs

The tissues of the salivary gland, fat body, Malpighian tubule, and midgut were dissected from third-instar *S. frugiperda*. Before dissection, the third-instar *S. frugiperda* was rinsed with 75% ethanol and dissected in chilled 1 × phosphate-buffered saline (pH 7.4) under a stereomicroscope. Each sample contained fifty insects and repeated three times. The tissues collected were kept and analyzed by RT-qPCR.

### miRNA Agomir Injection

The agomir-miR-278-5p, agomir-miR-13b-3p, agomir-miR-10485-5p, agomir-miR-10483-5p, and agomir-NC were synthesized in GenePharma (Shanghai, China). After anesthetized on ice, approximately 500 nl 20 μM agomir-NC, agomir-miR-278-5p, agomir-miR-13b-3p, agomir-miR-10485-5p, and agomir-miR-10483-5p were injected into the antesternum of third-instar *S. frugiperda* using a Nanoject II (Drummond, United States). The insects were treated with cyantraniliprole, spinetoram, and emamectin benzoate at the LC_50_ dose for 1 day after injection. All insects were reared at 25 ± 1°C with 50–70% humidity and 16 h light:8 h dark photoperiod. The mortality was checked after insecticide treatment in 24 h. Each treatment contained thirty insects and repeated three times.

### Statistical Analysis

SPSS 20.0 software (IBM Corporation, United States) was used to perform the statistical analyses. The Student’s *t*-test was used to compare the differences between treatments. Data were shown as mean ± standard error (SE). The *p* values <0.05 and 0.01 were considered statistically significant and very significant differences, respectively.

## Results

### Bioassay Evaluation

The toxicity of cyantraniliprole, spinetoram, and emamectin benzoate against the susceptible strain of *S. frugiperda* was tested. The LC_50_ values of cyantraniliprole, spinetoram, and emamectin benzoate were 0.304, 0.161, and 0.194 mg L^−1^, respectively (Table 1).

**TABLE 1:** Toxicity of insecticides to *S. frugiperda*.

Insecticide	LC_50_ (mg/L) (95% CL)	Slope±SE
Cyantraniliprole	0.304 (0.159–0.566)	1.162 ± 0.190
Spinetoram	0.161 (0.087–0.263)	0.799 ± 0.097
Emamectin benzoate	0.194 (0.124–0.285)	1.125 ± 0.139

### Illumina Sequencing Data in *S. frugiperda*

The sequencing data had been deposited in the Gene Expression Omnibus (GEO) at the National Center for Biotechnology Information (NCBI) with the accession number GSE189968. The twelve small RNA (sRNA) libraries derived from the control, cyantraniliprole-treated, spinetoram-treated, and emamectin benzoate-treated *S. frugiperda*. The total number of clean reads was 6,273,553, 11,856,426, 8,602,367, and 6,849,604 with unique reads of 1,252,300, 1,640,252, 1,173,199, and 1,478,489 from the cyantraniliprole treatment, spinetoram treatment, emamectin benzoate treatment, and the control libraries, respectively (Table 2). In the total reads, ribosomal RNAs (rRNAs) accounted for 64.66, 67.22, 68.61, and 72.79% among the non-miRNA sRNAs in the control, cyantraniliprole treatment, spinetoram treatment, and emamectin benzoate treatment libraries, respectively (Supplementary Figure S1). In the unique reads, rRNAs accounted for 69.63, 71.77, 70.17, and 70.72% in the control, cyantraniliprole treatment, spinetoram treatment, and emamectin benzoate treatment libraries, respectively (Supplementary Figure S1). The twelve sRNA libraries shared a similar length distribution pattern, with 22 nt being the most abundant (Supplementary Figure S2).

**TABLE 2:** Summary of small RNA sequencing.

	Control		Cyantraniliprole		Spinetoram		Emamectin benzoate	
	Total (%)	Unique (%)	Total (%)	Unique (%)	Total (%)	Unique (%)	Total (%)	Unique (%)
Adapter and length filter	30,479,744 (76.58)	3,562,960 (70.08)	16,518,185 (66.73)	2,825,331 (68.29)	12,351,507 (46.54)	2,737,311 (61.47)	16,080,835 (59.69)	3,059,536 (71.11)
Junk reads	51,468 (0.13)	8,723 (0.17)	40,076 (0.16)	7,476 (0.18)	58,586 (0.22)	9,920 (0.22)	36,674 (0.14)	7,118 (0.17)
Rfam	2,357,155 (5.92)	33,543 (0.66)	1,882,912 (7.61)	51,726 (1.25)	2,175,604 (8.20)	64,747 (1.45)	2,144,959 (7.96)	61,786 (1.44)
Repeats	63,636 (0.16)	327 (0.01)	39,984 (0.16)	542 (0.01)	95,187 (0.36)	674 (0.02)	76,019 (0.28)	679 (0.02)
Valid reads	6,849,604 (17.20)	1,478,489 (29.08)	6,273,553 (25.34)	1,252,300 (30.27)	11,856,426 (44.68)	1,640,252 (36.84)	8,602,367 (31.93)	1,173,199 (27.27)
Total reads	39,801,607 (100)	5,084,042 (100)	24,754,710 (100)	4,137,375 (100)	26,537,310 (100)	4,452,904 (100)	26,940,854 (100)	4,302,318 (100)

### Expression Profiles of miRNAs

**FIGURE 1:**
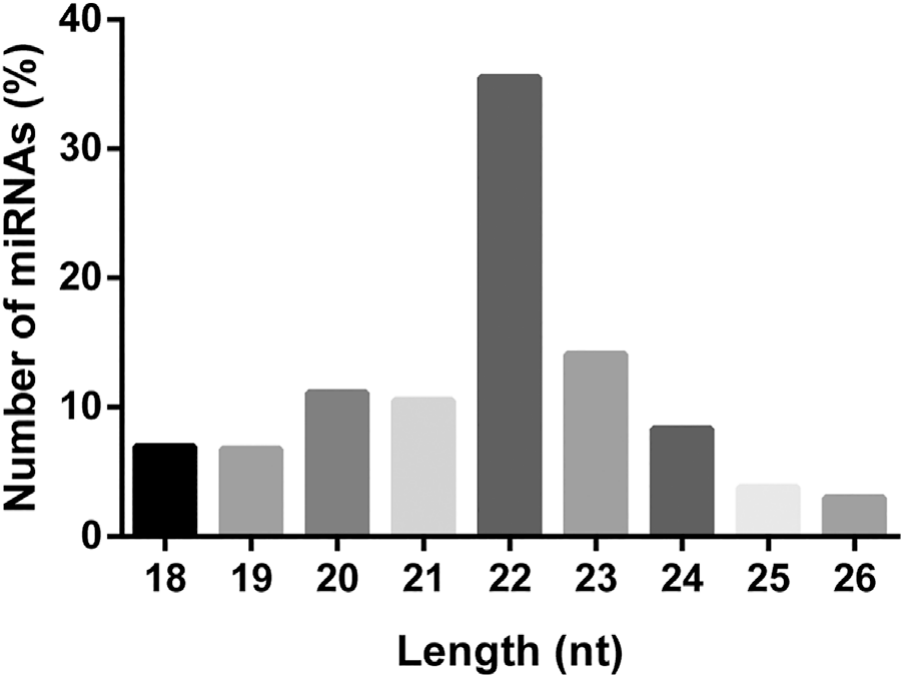
Length distribution of miRNAs in *S. frugiperda*.

**FIGURE 2:**
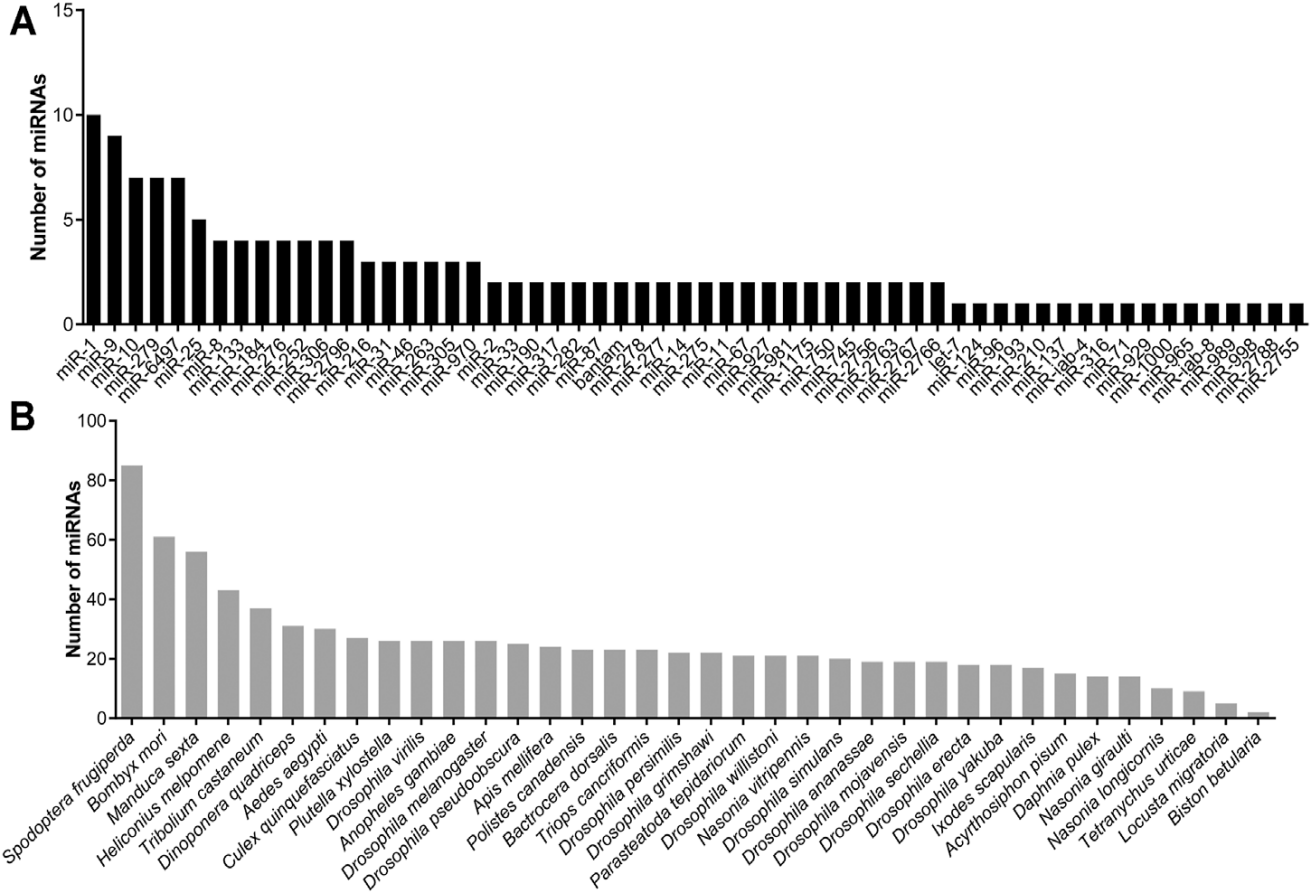
Characterization analysis of miRNAs in *S. frugiperda.* **(A)** Number of miRNAs in different family. **(B)** Number of miRNAs shared with other insect species.

A total of 504 miRNAs in *S. frugiperda* were identified, including 379 conserved miRNAs and 125 novel miRNAs (Supplementary Table S2). The length distribution of miRNAs in *S. frugiperda* was ranged from 18 nt to 26 nt, with 22 nt miRNAs being the most abundant, accounting for 35.52% (Figure 1). miR-1, miR-6497-5p, and miR-276-3p were the three most abundant miRNAs, and the information of the most highly expressed ten miRNAs is listed in Supplementary Table S3. The conservative profile of miRNAs in *S. frugiperda* was explored, and 58 miRNA sequence families were identified from the miRBase database (Figure 2A). miR-1 was the family with the largest number of miRNAs (Figure 2A). Furthermore, the frequency of miRNA identified in *S. frugiperda* in other insect species was analyzed. *Bombyx mori* and *Manduca sexta* shared 61 and 56 of their conserved miRNAs with *S. frugiperda*, respectively (Figure 2B). The sequencing data would greatly expand the number of miRNAs in *S. frugiperda*.

### Analysis of Differentially Expressed miRNAs

**FIGURE 3:**
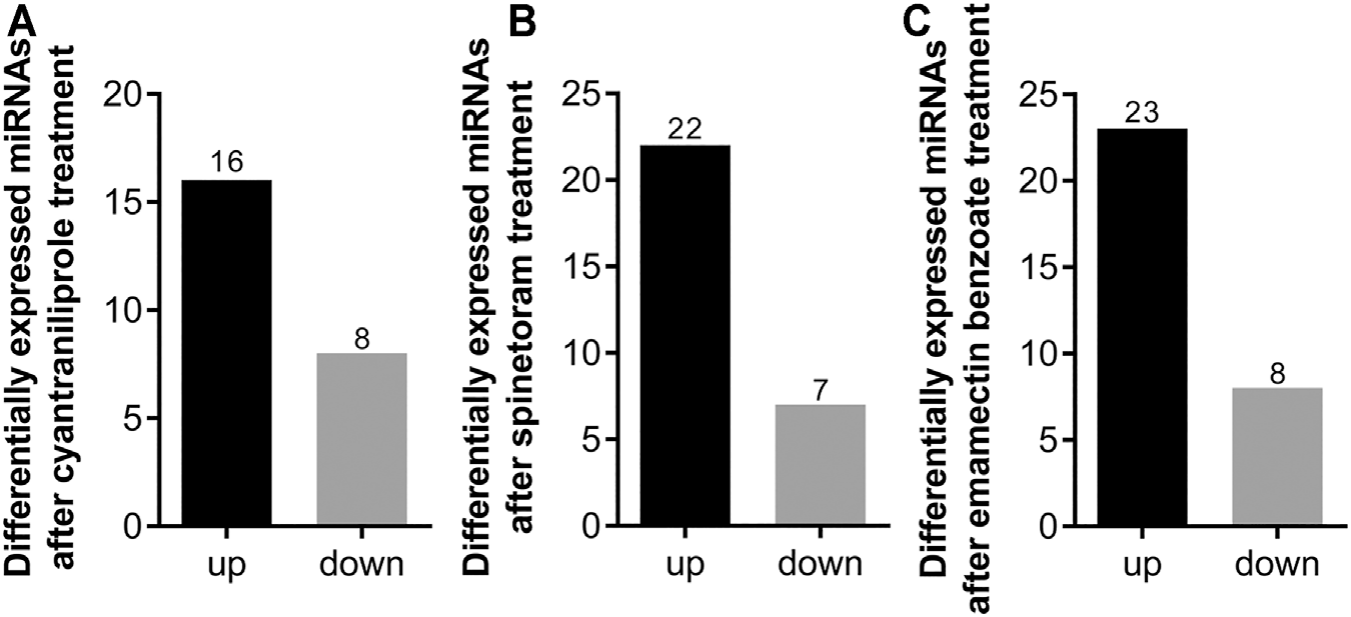
Number of differentially expressed miRNAs after insecticide treatment. **(A)** Number of differentially expressed miRNAs after cyantraniliprole treatment. **(B)** Number of differentially expressed miRNAs after spinetoram treatment. **(C)** Number of differentially expressed miRNAs after emamectin benzoate treatment.

After insecticide treatment, the differentially expressed miRNAs were determined by normalized read counts. Compared to control, 24 miRNAs were differentially expressed in cyantraniliprole treatment, 16 were significantly upregulated, and 8 were significantly downregulated (Figure 3A). A total of 22 miRNAs were significantly upregulated and 7 were significantly downregulated between spinetoram treatment and control (Figure 3B). In addition, 31 miRNAs were found to be differentially expressed in both emamectin benzoate treatment and control, 23 were upregulated, and 8 were downregulated (Figure 3C). These results indicated that miRNAs could be induced by insecticides in *S. frugiperda*.

### Prediction Targets of Differentially Expressed miRNAs and Functional Analysis

**FIGURE 4:**
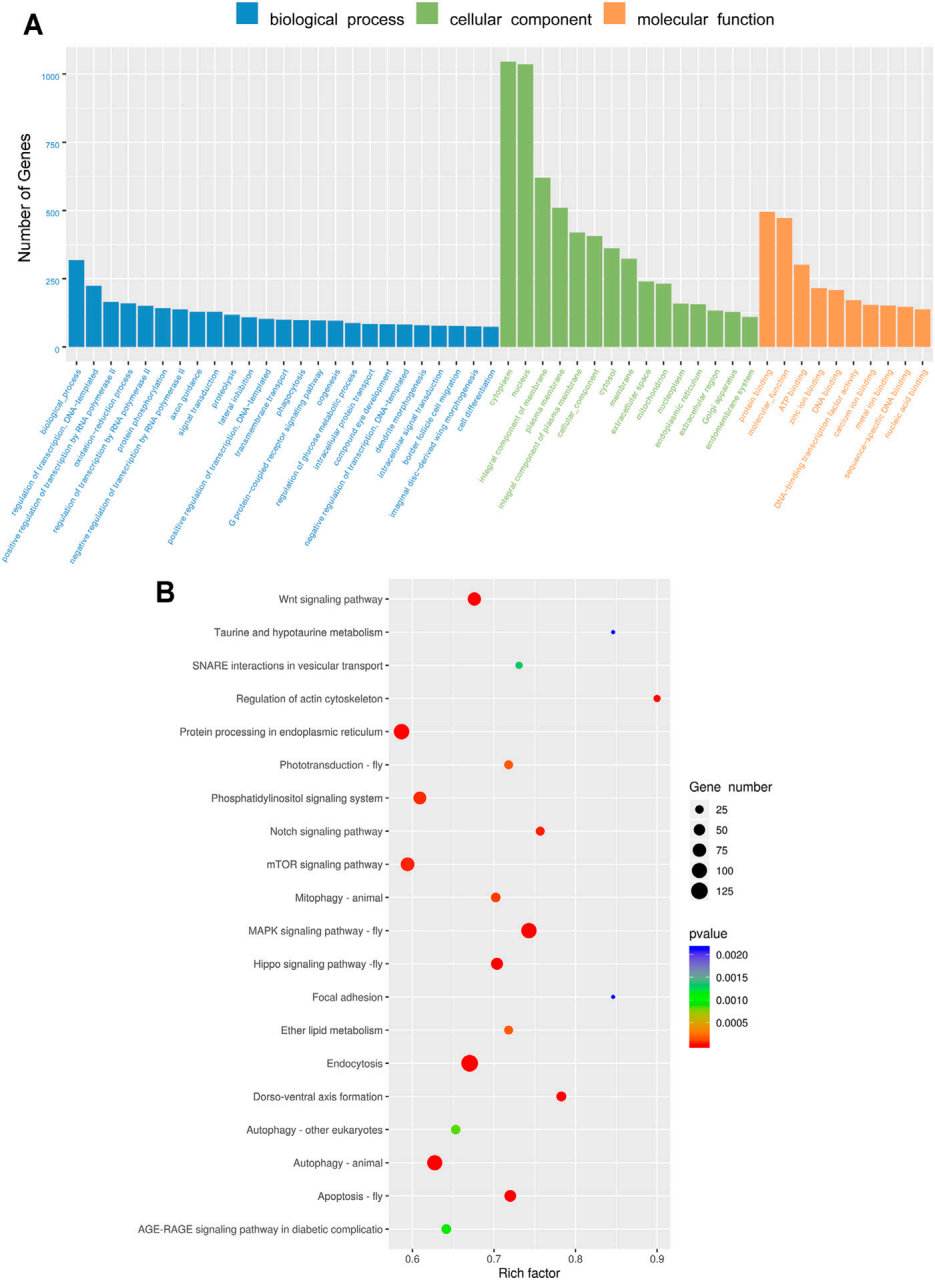
Gene Ontology (GO) term and the Kyoto Encyclopedia of Genes and Genomes (KEGG) pathway of the target genes. **(A)** Gene Ontology (GO) term of the target genes of differentially expressed miRNAs. **(B)** Kyoto Encyclopedia of Genes and Genomes (KEGG) pathway enrichment of the target genes of differentially expressed miRNAs.

**FIGURE 5:**
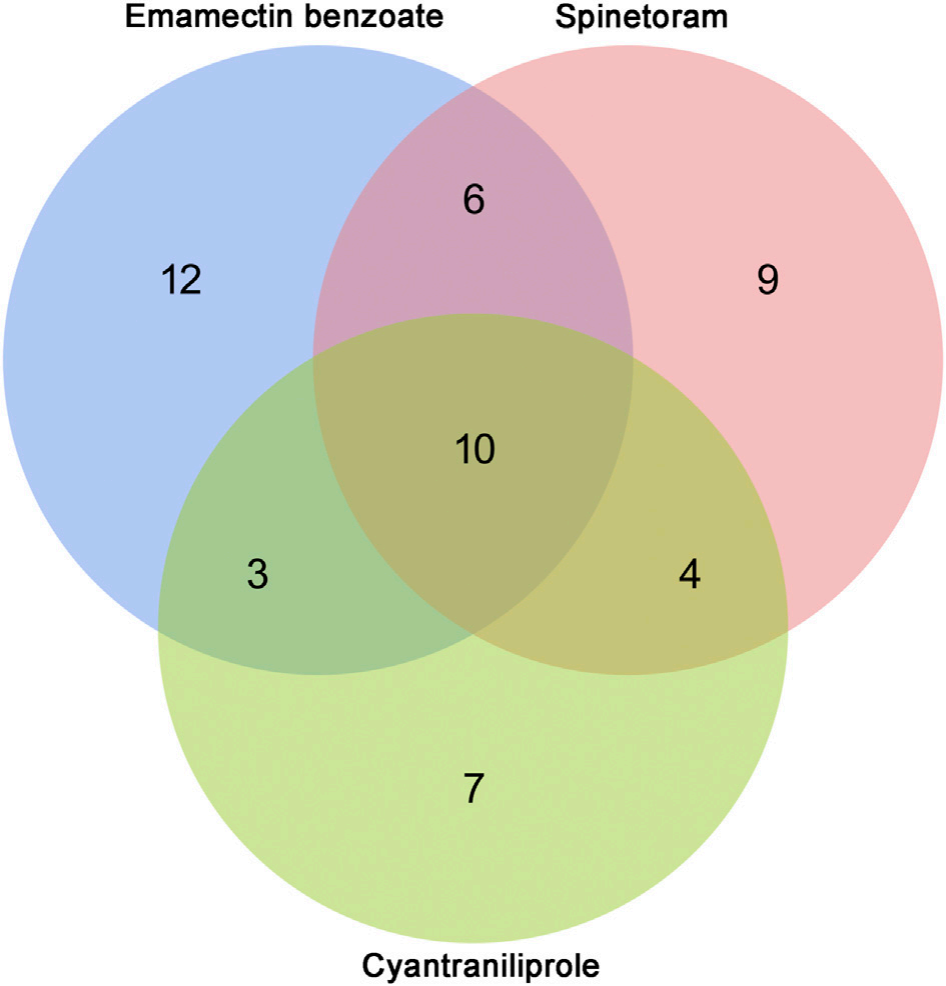
Venn diagram of differentially expressed miRNAs after cyantraniliprole, spinetoram, and emamectin benzoate treatment.

In order to understand the miRNA functions in insecticide resistance, the targets of differentially expressed miRNAs were predicted (Supplementary Table S4). To predict the functions of these target genes, the GO and KEGG enrichment analyses were performed. The target genes were assigned the terms of the biological process, cellular component, and molecular function, and the cytoplasm was the most enriched subgroup (Figure 4A). The top twenty significantly enriched KEGG pathways of the miRNA target genes are presented in Figure 5. The MAPK signaling pathway-fly, endocytosis, Wnt signaling pathway, autophagy-animal, and apoptosis-fly were the five most significantly enriched pathways (Figure 4B).

### Analysis of Common Differentially Expressed miRNAs Under Insecticide Treatment

**FIGURE 6:**
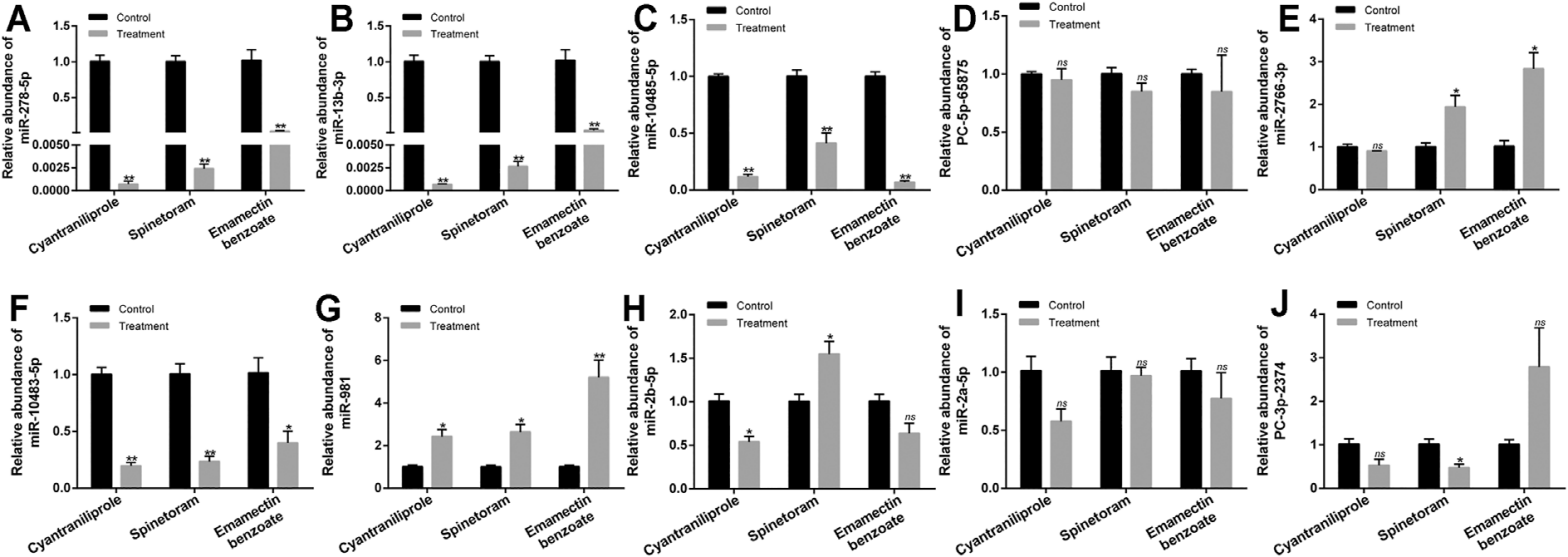
Relative expression of ten miRNAs after the treatments of cyantraniliprole, spinetoram, and emamectin benzoate. **(A–J)** Relative expressions of miR-278-5p, miR-13b-3p, miR-10485-5p, PC-5p-65875, miR-2766-3p, miR-10483-5p, miR-981, miR-2b-5p, miR-2a-5p, and PC-3p-2374 after the treatments of cyantraniliprole, spinetoram, and emamectin benzoate. The asterisk (*) and asterisks (**) indicate *p* < 0.05 and *p* < 0.01. *ns* represents no significant difference. Results are shown as the average±standard error (SE).

A total of ten miRNAs were significantly differentially expressed among the treatments of cyantraniliprole, spinetoram, and emamectin benzoate, including miR-278-5p, miR-13b-3p, miR-10485-5p, PC-5p-65875, miR-2766-3p, miR-10483-5p, miR-981, miR-2b-5p, miR-2a-5p, and PC-3p-2374 (Supplementary Table S5, Figure 5). The relative expression of ten differentially expressed miRNAs was validated by RT-qPCR. Compared to control, 4 miRNAs, including miR-278-5p, miR-13b-3p, miR-10485-5p, and miR-10483-5p, were significantly downregulated among the treatments of cyantraniliprole, spinetoram, and emamectin benzoate (Figures 6A–C,F). miR-981 was significantly upregulated among the treatments of cyantraniliprole, spinetoram, and emamectin benzoate (Figure 6G). miR-2766-3p was significantly upregulated between spinetoram and emamectin benzoate treatments (Figure 6E). miR-2b-5p was significantly downregulated and upregulated after cyantraniliprole and spinetoram treatments, respectively (Figure 6H). PC-3p-2374 was significantly downregulated after the spinetoram treatment (Figure 6J). The relative expression levels of PC-5p-65875 and miR-2a-5p showed no significant difference among the treatments of cyantraniliprole, spinetoram, and emamectin benzoate compared to control (Figures 6D–I).

**FIGURE 7:**
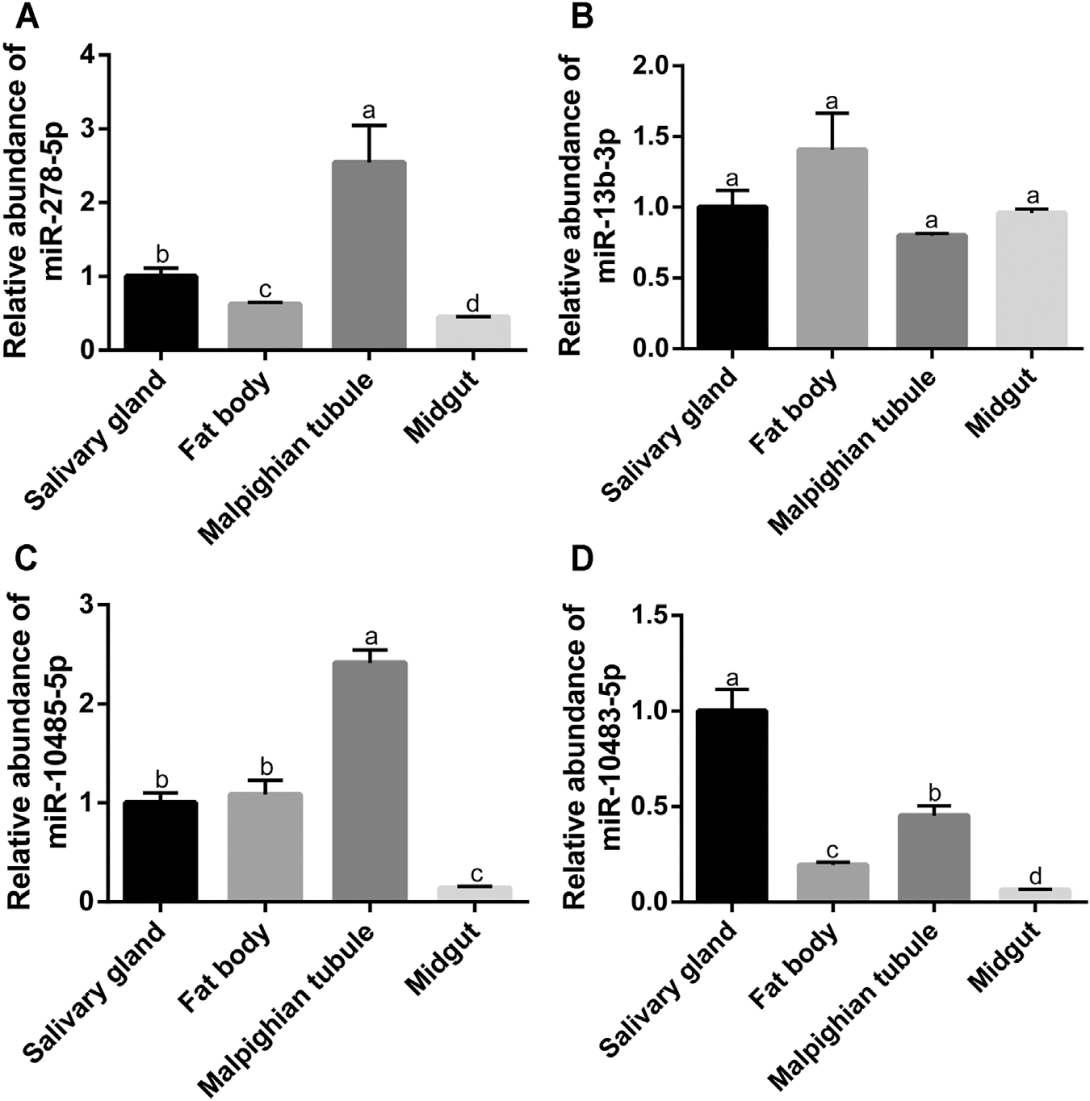
Expression of four miRNAs in different tissues of *S. frugiperda*. **(A–D)** Analysis of the miR-278-5p expression at the different tissues of the salivary gland, fat body, Malpighian tubule, and midgut. The letters a, b, c, d, e, and f represent significant differences (*p* < 0.05). Results are shown as the average±standard error (SE).

Furthermore, the expression profiles of miR-278-5p, miR-13b-3p, miR-10485-5p, and miR-10483-5p were further investigated in different tissues of *S. frugiperda*. As shown in Figure 7A, miR-278-5p had the highest expression in the Malpighian tubule and the lowest expression in the midgut. The expression of miR-13b-3p had no significant difference in the tissues of the salivary gland, fat body, Malpighian tubule, and midgut (Figure 7B). Similar to miR-278-5p, miR-10485-5p had the highest expression in the Malpighian tubule and the lowest expression in the midgut (Figure 7C). The expression of miR-10483-5p had the highest expression in the salivary gland and the lowest expression in the midgut (Figure 7D).

The predicted target genes of miR-278-5p, miR-13b-3p, miR-10485-5p, and miR-10483-5p that might participate in the xenobiotic metabolism were further investigated. Some target genes have been verified to play vital functions in responses to xenobiotics, such as G-protein–coupled receptor, cytochrome P450 monooxygenases, ATP-binding cassette transporters, and UDP-glycosyltransferase (Supplementary Table S6).

### miRNAs Impact the Susceptibility of *S. frugiperda* to Insecticides

**FIGURE 8:**
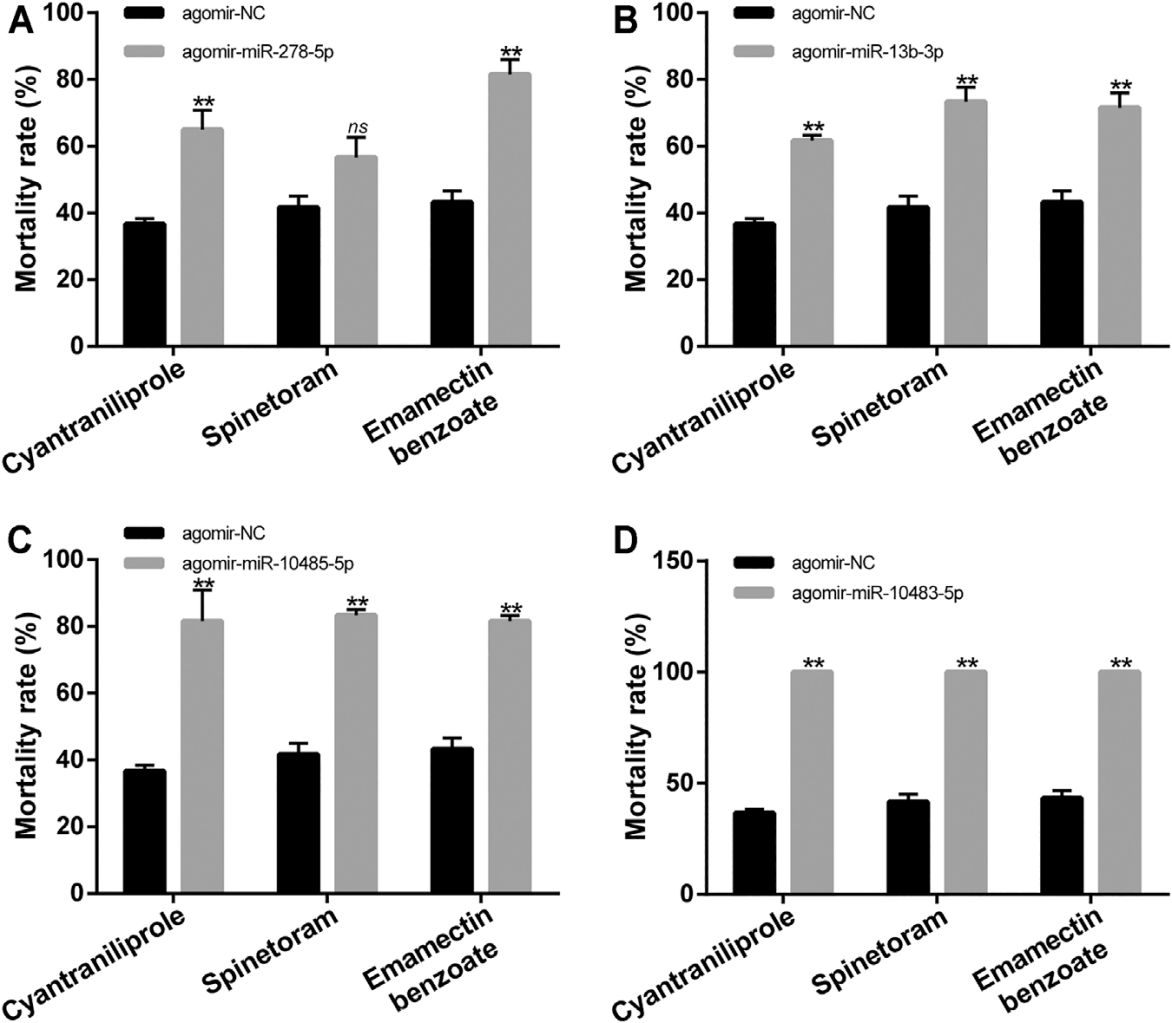
Mortality of *S. frugiperda* with LC_50_ doses of cyantraniliprole, spinetoram, and emamectin benzoate treatments after injection of miRNA agomir. **(A)** Mortality of *S. frugiperda* with LC_50_ doses of treatment of three insecticides after injection of agomir-miR-278-5p. **(B)** Mortality of *S. frugiperda* with LC_50_ doses of treatment of three insecticides after injection of agomir-miR-13b-3p. **(C)** Mortality of *S. frugiperda* with LC_50_ doses of treatment of three insecticides after injection of agomir-miR-10485-5p. **(D)** Mortality of *S. frugiperda* with LC_50_ doses of treatment of three insecticides after injection of agomir-miR-10483-5p. The asterisks (**) indicate *p* < 0.01. *ns* represents no significant difference. Results are shown as the average±standard error (SE).

The functions of miR-278-5p, miR-13b-3p, miR-10485-5p, and miR-10483-5p in the tolerance of cyantraniliprole, spinetoram, and emamectin benzoate were studied by miRNA agomir injection. The relative abundance of miR-278-5p, miR-13b-3p, miR-10485-5p, and miR-10483-5p was significantly elevated by injection of agomir-miR-278-5p, agomir-miR-13b-3p, agomir-miR-10485-5p, and agomir-miR-10483-5p (Supplementary Figure S3). The mortality was significantly increased in agomir-miR-278-5p, agomir-miR-13b-3p, agomir-miR-10485-5p, and agomir-miR-10483-5p injection, with LC_50_ doses of cyantraniliprole and emamectin benzoate treatment (Figures 8A–D). In addition, the mortality was significantly increased in agomir-miR-13b-3p, agomir-miR-10485-5p, and agomir-miR-10483-5p injection, with an LC_50_ dose of spinetoram treatment (Figures 8B–D). The mortality showed no significant difference in agomir-miR-278-5p injection, with an LC_50_ dose of spinetoram treatment (Figure 8A). These results indicated that miR-278-5p, miR-13b-3p, miR-10485-5p, and miR-10483-5p could regulate the tolerance of *S. frugiperda* to insecticides.

## Discussion

With the advent of new computational methods and deep-sequencing technologies, an increasing number of miRNAs have been identified in several eukaryotes (Asgari, 2013). The functions of miRNAs are mainly focused on a variety of physiological processes in insects (Lucas et al., 2015b). Recently, major progress has been made in the miRNA regulatory roles in insecticide resistance by targeting detoxification genes in several insect species (Zhang et al., 2021b). It has been reported that a series of miRNAs participate in the antiviral immune defense in *S. frugiperda* (Karamipour et al., 2019). However, the profiles and functions of miRNAs in insecticide resistance and detoxification are still unknown in *S. frugiperda*.

Our present research systematically identified the miRNAs that might play regulatory roles in the tolerance of cyantraniliprole, spinetoram, and emamectin benzoate in *S. frugiperda*. A total of 379 conserved and 125 novel miRNAs existing in *S. frugiperda* were systematically identified in this study. A previous study has identified 215 and 239 miRNAs in the “corn-strain” and “rice-strain” *S. frugiperda*, respectively (Moné et al., 2018). Our sequencing data provide an important basis for further research about miRNAs in different physiological processes. The number of miRNAs identified in *S. frugiperda* was similar to that of other insects, such as *Plutella xylostella* (Zhu et al., 2017). The 22 nt length was the most abundant miRNAs in *S. frugiperda* which was similar to the previous reports in insects (Zhang et al., 2021c; Li et al., 2015b; Zhu et al., 2017; Mao et al., 2021; Zhang et al., 2021a). In addition, miR-1, miR-6497-5p, and miR-276-3p were the three most abundant miRNAs in *S. frugiperda*. miR-1 is a widely studied conserved miRNA, and a recent research showed that miR-1-3p was significantly downregulated in cyflumetofen-resistant strain of *Tetranychus cinnabarinus* and involved in the resistance to cyflumetofen *via* targeting the glutathione S-transferase gene (Zhang et al., 2018). These results indicated that the high abundance of miRNAs identified in *S. frugiperda* might also participate in the insecticide tolerance.

A total of 24, 22, and 31 miRNAs were differentially expressed after treatments of cyantraniliprole, spinetoram, and emamectin benzoate, respectively, suggesting that these miRNAs might play a regulatory role in the detoxification of insecticides in *S. frugiperda*. miRNAs play critical roles by regulating the transcript levels of target mRNA genes, thereby understanding the functions of target genes will be helpful to reveal the roles of miRNAs. In this study, the target genes of differentially expressed miRNAs were predicted by GO and KEGG enrichment analyses. Interestingly, the MAPK signaling pathway was the most significantly enriched pathway. Recent research has shown that cyantraniliprole could regulate the reproduction of *Bactrocera dorsalis* by affecting 20-hydroxyecdysone through the MAPK signaling pathway (Li et al., 2021b). These results indicated that miRNAs could affect the physiological and developmental pathways of *S. frugiperda* by the MAPK signaling pathway.

Furthermore, we found the relative expressions of miR-278-5p, miR-13b-3p, miR-10485-5p, PC-5p-65875, miR-2766-3p, miR-10483-5p, miR-981, miR-2b-5p, miR-2a-5p, and PC-3p-2374 were all significantly differentially expressed among the treatments of cyantraniliprole, spinetoram, and emamectin benzoate. The common differentially expressed miRNAs were likely to be involved in the tolerance of cyantraniliprole, spinetoram, and emamectin benzoate. However, as insects have complex insecticide-resistant mechanisms, these 10 miRNAs may also reveal common mechanisms of resistance to these three insecticides. Previous reports have shown that miR-2 and miR-13 could regulate the resistance to deltamethrin in *Culex pipiens pallens* (Guo et al., 2017). In addition, miR-278-3p could regulate the resistance to pyrethroid in *C. pipiens pallens* by targeting *CYP6AG11* (Lei et al., 2015). In order to further explore the roles of these 10 miRNAs in the tolerance of insecticides, the relative expressions were detected in *S. frugiperda* after treatments of cyantraniliprole, spinetoram, and emamectin benzoate by RT-qPCR. miR-278-5p, miR-13b-3p, miR-10485-5p, and miR-10483-5p were all significantly downregulated among the treatments of these three insecticides. Through tissue expression profiling, we found miR-278-5p, miR-10485-5p, and miR-10483-5p that were lowly expressed in the midgut of *S. frugiperda*, suggesting that their target genes were highly expressed in the midgut. Some target genes of miR-278-5p, miR-13b-3p, miR-10485-5p, and miR-10483-5p which previously reported relevant to insecticide resistance were discovered, mainly including P450s, ABC transporters, and UDP-glycosyltransferase (Lin et al., 2013; Perreault et al., 2013; Meng et al., 2020). However, a relationship between miRNAs and their target genes needs to be further researched. These results indicated that miRNAs might regulate the resistance to insecticides in *S. frugiperda* by targeting genes putatively participated in insecticide detoxication.

To further reveal the functions of miRNAs in the insecticide tolerance, we analyzed the mortality of *S. frugiperda* to cyantraniliprole, spinetoram, and emamectin benzoate after injection of miRNA agomir. The overexpression of miR-278-5p, miR-13b-3p, miR-10485-5p, and miR-10483-5p significantly increased the mortality of *S. frugiperda* to cyantraniliprole and emamectin benzoate treatment. Furthermore, the mortality was significantly increased with spinetoram treatment after overexpression of miR-13b-3p, miR-10485-5p, and miR-10483-5p. These results indicated that miR-278-5p, miR-13b-3p, miR-10485-5p, and miR-10483-5p could regulate the tolerance of *S. frugiperda* to cyantraniliprole, spinetoram, and emamectin benzoate and indicated that these four miRNAs might reveal a common mechanism to insecticide resistance.

## Conclusion

In summary, we identified the differentially expressed miRNAs in *S. frugiperda* after cyantraniliprole, spinetoram, and emamectin benzoate treatments. The effects of miR-278-5p, miR-13b-3p, miR-10485-5p, and miR-10483-5p on the tolerance of *S. frugiperda* to cyantraniliprole, spinetoram, and emamectin benzoate were analyzed. These results indicate that miRNAs play key roles in the resistance of *S. frugiperda* to insecticides.

## Data Availability

The original contributions presented in the study are publicly available. This data can be found here: National Center for Biotechnology Information (NCBI) BioProject database under accession number GSE189968.
