# Supplementary figures and images for: Four MicroRNAs, miR-13b-3p, miR-278-5p, miR-10483-5p, and miR-10485-5p, Mediate Insecticide Tolerance in Spodoptera frugiperda
Source: Front Genet. 2022 Jan 21;12:820778. doi: 10.3389/fgene.2021.820778 (PMC8814628; doi:10.3389/fgene.2021.820778)

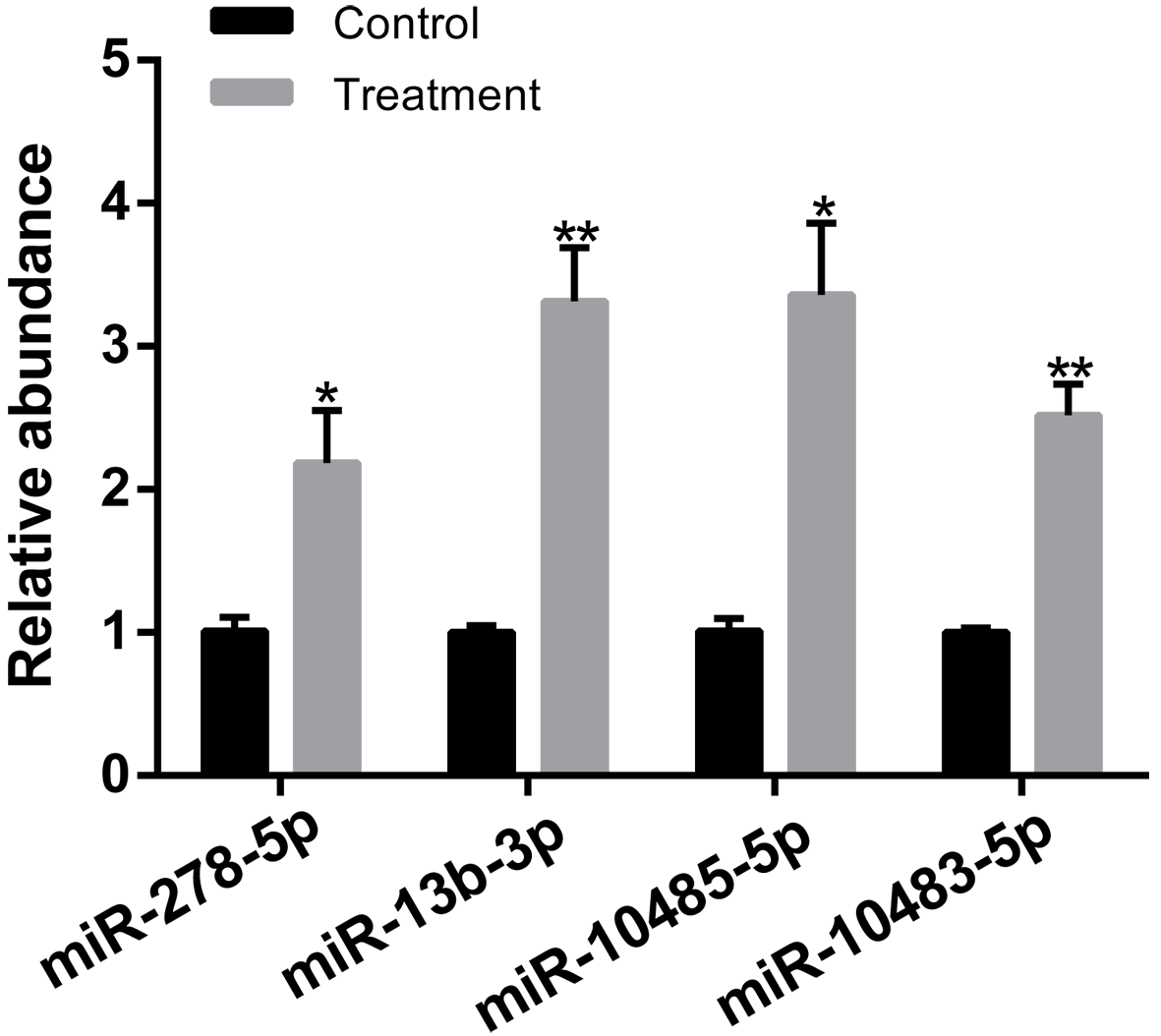

Supplement: Supplementary file 3 [file Image3.TIF]

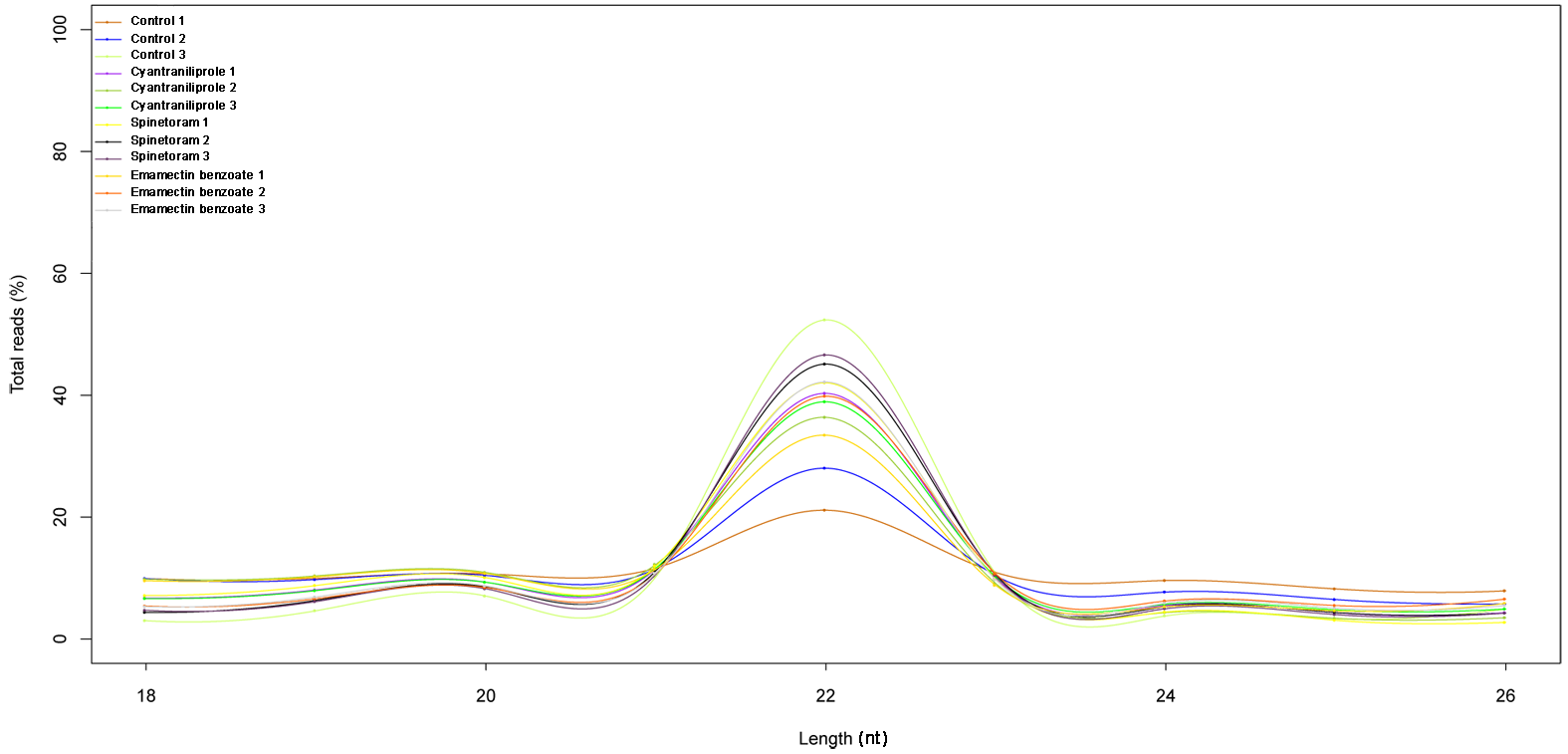

Supplement: Supplementary file 4 [file Image2.TIF]

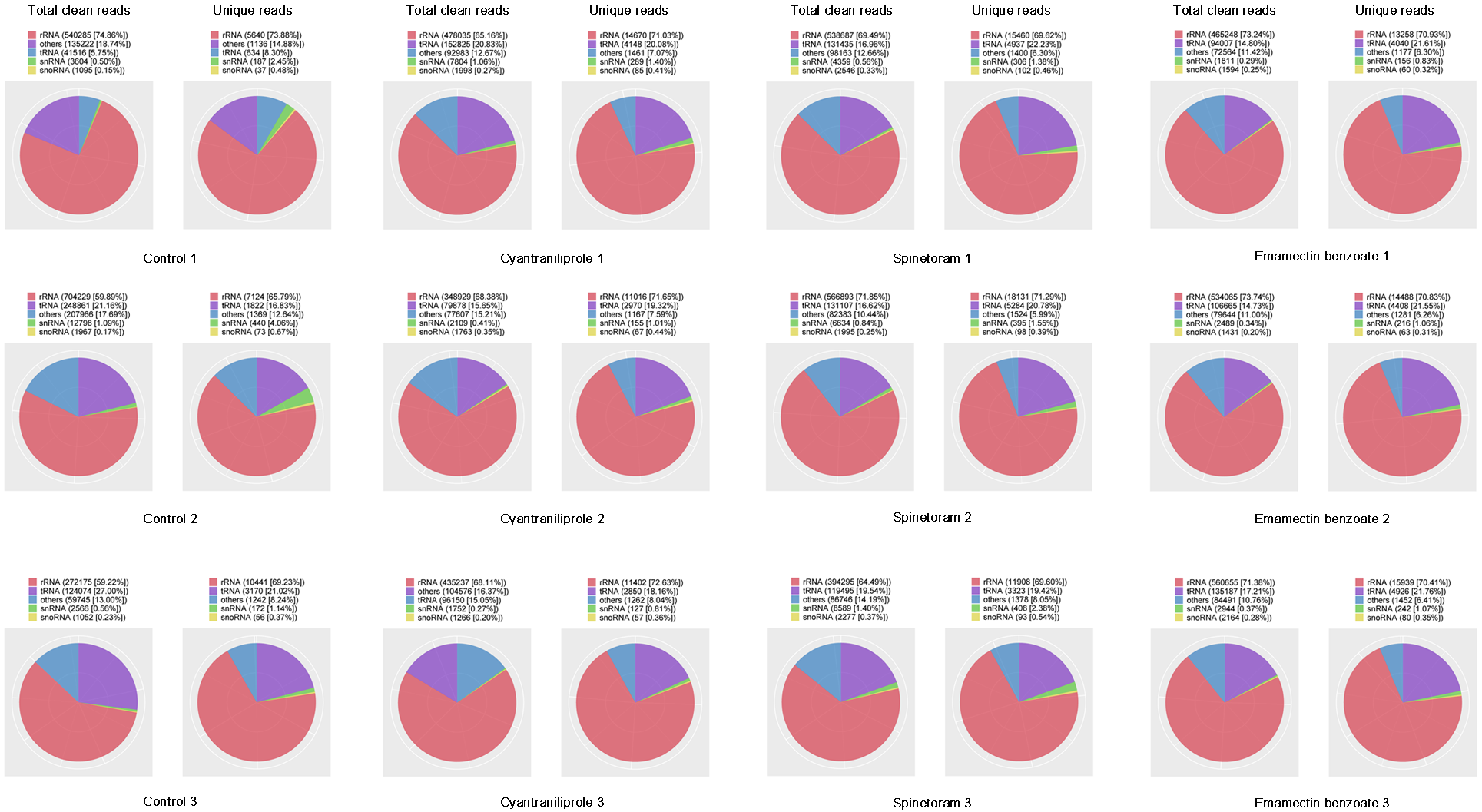

Supplement: Supplementary file 5 [file Image1.TIF]
